# Supplementary material for: Adipocyte adaptive immunity mediates diet-induced adipose inflammation and insulin resistance by decreasing adipose Treg cells
Source: Nat Commun. 2017 Jul 12;8:15725. doi: 10.1038/ncomms15725 (PMC5510177; doi:10.1038/ncomms15725)
Supplement: Supplementary Information — Supplementary figures [file ncomms15725-s1.pdf]

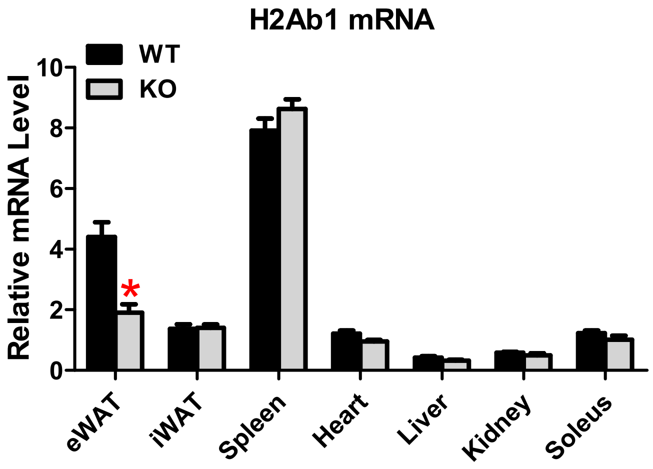

Supplementary figure 1. H2Ab1 mRNA expression in tissues (N=6/group) from chow-fed mice. The data was presented as relative mRNA levels. Mean±SE; *p<0.05 vs. WT by T-test.

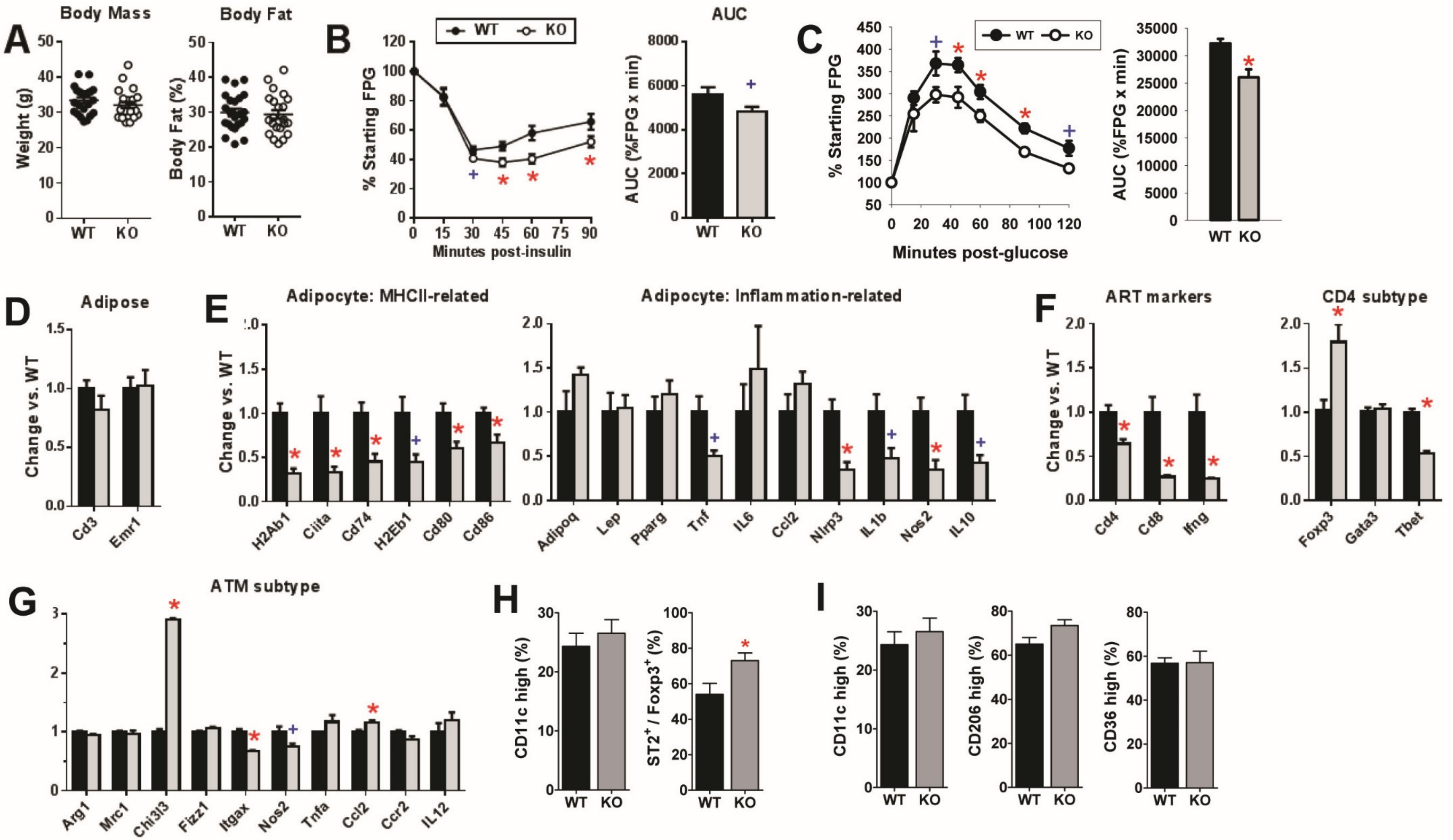

Supplementary figure 2. Adipocyte MHCII deficiency attenuates insulin resistance and adipose inflammation after 6 weeks of HFD. A) Body mass and adiposity (N=22-23/group) and **B)** intraperitoneal insulin tolerance test (N=14-17/group) and **C)** intraperitoneal glucose tolerance test (N=6-8/group) of 6 week HFD-fed WT and aMHCII$^{-/-}$ mice. The area under the curve (AUC) was measured in each case. Gene expression profiles of epididymal **D)** adipose tissue, **E)** adipocytes and **F)** ARTs and **G)** ATMs after 6 weeks HFD (N=3-5/group). **H)** Flow cytometry analysis of Treg and ST2^{+} Treg in adipose CD4^{+} T cells (N=4/group) and **I)** flow cytometry analysis of CD11c, CD206 and CD36 in ATMs (N=4-6/group) from 6 week HFD-fed WT and aMHCII$^{-/-}$ mice. Mean±SE; *p<0.05 or +p<0.1 vs. WT by T-test.

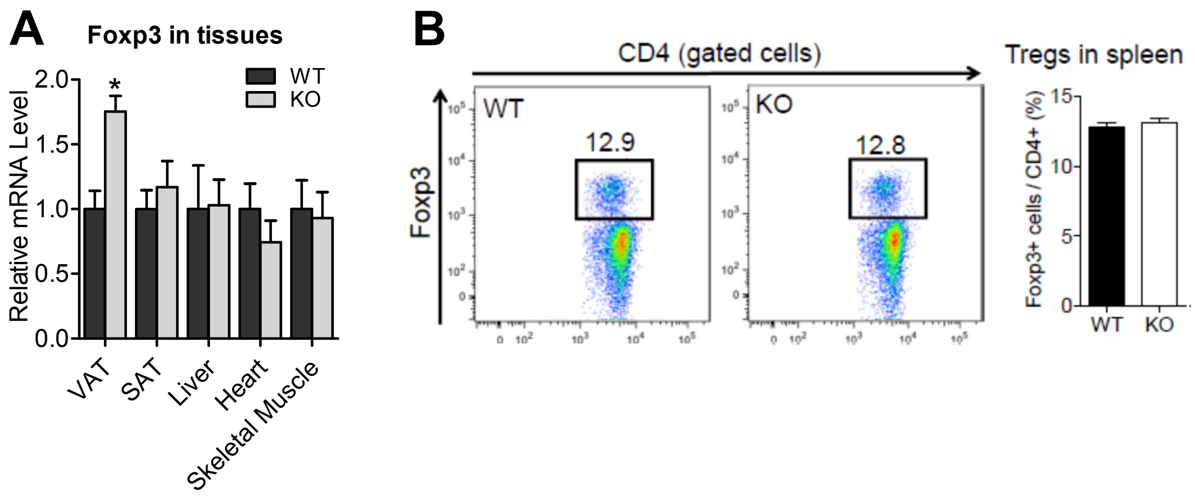

Supplementary figure 3. Foxp3 expression in metabolic tissues and spleen. A) Foxp3 expression in tissues of wild-type (WT) and aMHCII$^{-/-}$ (KO) mice at 12 weeks HFD (N=4/group). **B)** Flow cytometry analysis of Foxp3^{+} Tregs in spleens from wild-type (WT) and aMHCII$^{-/-}$ (KO) mice at 12 weeks HFD (N=3/group). Mean±SE; *p<0.05 vs. WT by T-test.

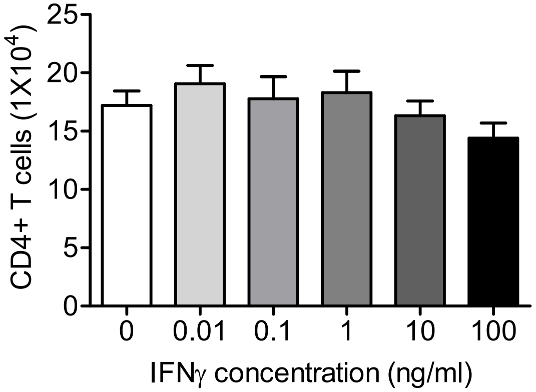

Supplementary figure 4. IFNγ did not alter T cell number in Treg differentiation assays. Treg differentiation in naïve splenic T cell cultures supplemented with different concentrations of recombinant IFNγ. Total $CD4^+$ T cells were counted four days after initiation of Treg differentiation. Mean±SE; N=4/group.

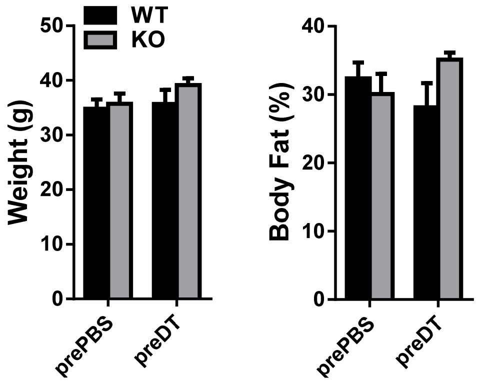

Supplementary figure 5. Body weight and adiposity of WT and aMHCII$^{-/-}$ Foxp3-DTR BMT mice. WT and aMHCII$^{-/-}$ Foxp3-DTR BMT cohorts reveal similar body mass and adiposity at 10 weeks HFD prior to DT treatment. Mean±SE; N=6-9/group.

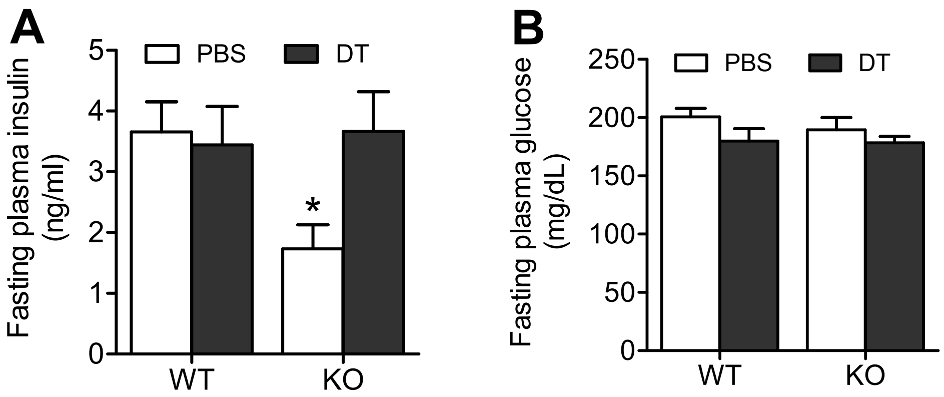

Supplementary figure 6. Treg ablation normalizes the difference in fasting plasma insulin between obese WT and aMHCII$^{-/-}$ mice. WT and aMHCII KO mice were fed on HFD and treated with PBS or DT during weeks 10-14 of HFD. The plasma samples were collected from 6h-fasted mice. The plasma insulin **A)** was measured by ELISA from EMD Millipore. The plasma glucose **B)** was measured by OneTouch glucose meter. (Mean±SE; N=6-9/group; *p<0.05 vs. WT or genotype-matched DT by T -test.).

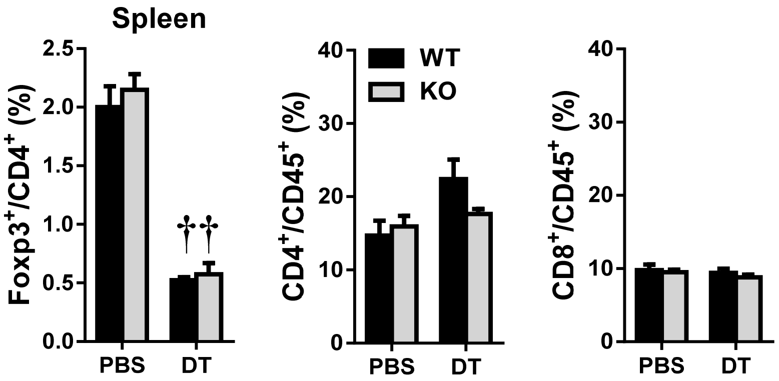

Supplementary figure 7. Treg ablation does not otherwise alter splenic CD4^{+} and CD8^{+} T cell populations. FACS analysis of splenic T cell subtypes in HFD-fed after 4 weeks of PBS or DT treatment. Mean±SE; N=4/group; $p<0.05$ vs. WT(*) or PBS(†)

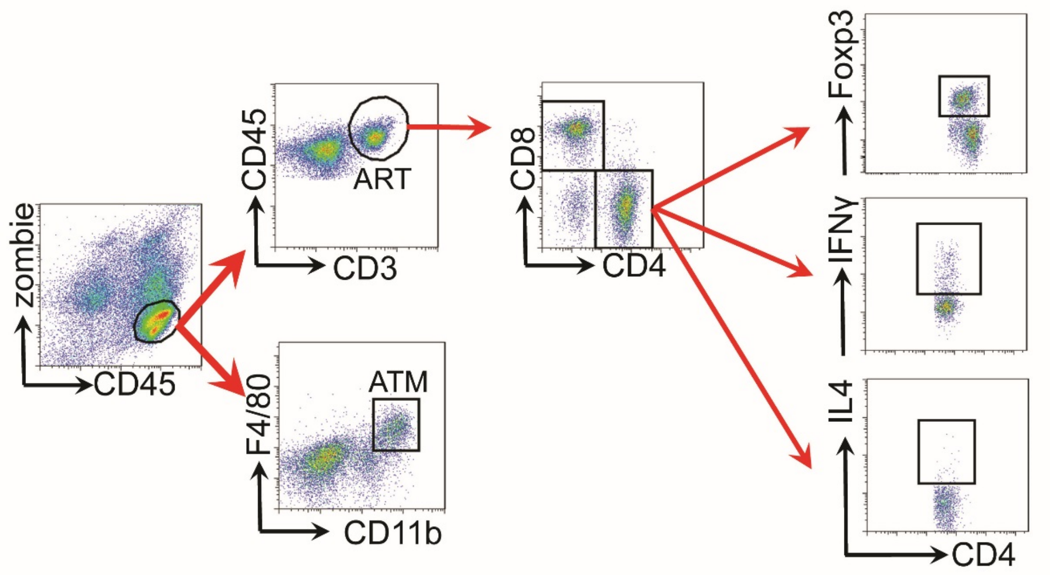

Supplementary figure 8. Gating of T cell and macrophage populations in SVF. CD45$^+$ and zombie- population was gated as viable leukocytes. CD3$^+$ cells in live leukocytes (adipose resident T cell, ART) were separated into CD8$^+$ and CD4$^+$ fractions. The CD4$^+$ cells were further analyzed for Foxp3$^+$ Tregs. F4/80$^+$CD11b$^+$ cells in live leukocytes were gated as adipose tissue macrophage (ATM) for further analysis.

WAT

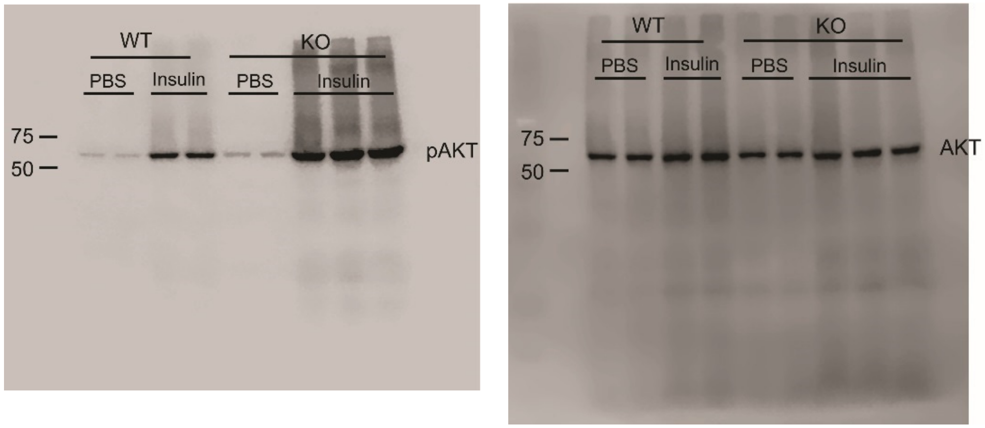

Skeletal Muscle

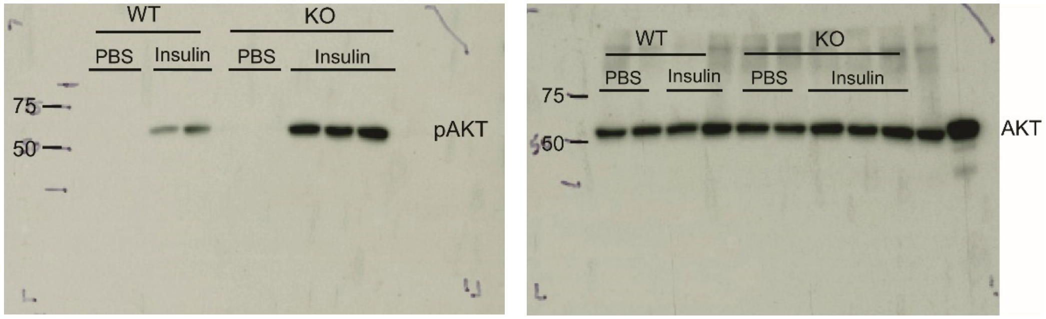

Supplementary figure 9. Immunoblots in Fig. 2e.
